# Antiviral activity of eicosapentaenoic acid against zika virus and other enveloped viruses

**DOI:** 10.3389/fphar.2025.1564504

**Published:** 2025-04-04

**Authors:** Yifei Feng, Shuqi Qiu, Shuting Zou, Ru Li, Hongyu Chen, Kaitian Chen, Junbo Ma, Jinyu Liu, Xiaoyun Lai, Shuwen Liu, Min Zou

**Affiliations:** ^1^ NMPA Key Laboratory for Research and Evaluation of Drug Metabolism, School of Pharmaceutical Sciences, Southern Medical University, Guangzhou, China; ^2^ Shenzhen Luohu District People’s Hospital, Shenzhen, China; ^3^ School of Traditional Chinese Medicine, Southern Medical University, Guangzhou, China; ^4^ Guangdong Provincial Key Laboratory of New Drug Screening, School of Pharmaceutical Sciences, Southern Medical University, Guangzhou, China

**Keywords:** ZIKV, EPA, antiviral activity, binding, adsorption, entry

## Abstract

**Background:**

Zika virus (ZIKV) is an emerging flavivirus that may cause innate microcephaly or neurological disturbances. Yet no antiviral has been approved by FDA against ZIKV infection. It was shown that some unsaturated fatty acids could inactivate enveloped viruses including SARS-CoV-2. However, studies investigating the effect of eicosapentaenoic acid (EPA) on ZIKV infection are lacking. This study aims to evaluate the antiviral effect of EPA against ZIKV and other enveloped viruses.

**Methods:**

We first explored the toxicities of EPA *in vitro* and *in vivo*. Then we examined the antiviral effect of EPA against ZIKV via cell-based immunodetection, qRT-PCR, Western blotting, and so on. To uncover its antiviral mechanism, we performed assays for virus binding, adsorption and entry, and time-of-addition. RNase digestion and ZIKV NS2B-NS3 protease inhibition assays were also adopted. Finally, we detected its effects on dengue virus (DENV)-2, herpes simplex virus (HSV)-1 and influenza A virus via MTT, Western blotting and qRT-PCR assays.

**Results:**

EPA was found to inhibit ZIKV infection *in vitro* without causing cytotoxicities. EPA exhibited antiviral activity in the early stages of the ZIKV life cycle quickly. Mechanistic experiments showed that EPA disrupted the membrane integrity of viral particles, leading to the release of viral RNA, together with the interruption of ZIKV from binding, adsorption and entry, and ultimately the inhibition of viral proliferation. Furthermore, EPA exerted antiviral effects against DENV-2, HSV-1, and influenza virus, in a dose-dependent manner.

**Conclusion:**

These findings suggest that EPA is a promising broad-spectrum antiviral drug candidate.

## 1 Introduction

Zika virus (ZIKV) is an emerging pathogen that is mainly transmitted to humans through mosquito vectors. Although ZIKV infection is usually asymptomatic or self-limiting, it sometimes causes neurological complications (Koppolu and Shantha Raju, 2018; Turpin et al., 2022). Owing to the explosive outbreak in 2015 and its association with severe congenital birth defects, World Health Organization (WHO) declared it a public health emergency on 1 February 2016 (Bhagat et al., 2021; Bell et al., 2016). It was validated in cell and mouse models that ZIKV infection could lead to excessive cell death in the developing brain *in utero*, resulting in various motor and cognitive disorders in newborns (Pena et al., 2018; Victorio et al., 2024; Jagger et al., 2017). And the infection in susceptible male mice also caused testis damage and infertility (Ma et al., 2016). Numerous studies have been conducted to develop vaccines or drugs against ZIKV infection, but up till now none has been approved due to limited solubility, poor stability, or susceptibility to degradation by proteases in the body (Parker et al., 2021). Therefore, developing effective and accurate targeted drugs for treating ZIKV infection is necessary.

ZIKV is an enveloped particle with a diameter of approximately 50 nm and a genome comprising a single-stranded positive-sense RNA molecule of approximately 11 kb in size (Sirohi et al., 2016; Peregrine et al., 2019). For all flaviviruses, the RNA genome encodes three structural genes (C, M, and E) and seven non-structural genes (NS1, NS2A, NS2B, NS3, NS4A, NS4B, and NS5), with untranslated regions (UTRs) being located at the 5′and 3′ends (Shi and Gao, 2017; Hamel et al., 2015). The outer viral membrane is a lipid bilayer containing viral membrane (M) and envelope (E) proteins. In particular, glycosylation of E protein is a decisive factor for virus invasion into nerves (Neal, 2014; DiNunno et al., 2020). In mature flaviviruses, the elementary unit of E protein is a dimer in which each E monomer shares a 2-fold symmetry with its neighbor (Kostyuchenko et al., 2016; Hu et al., 2021). Seven C-terminal non-structural proteins are essential for the replication of viral RNA. Two non-structural proteins, NS2B-NS3 and NS5, are the main catalysts for viral RNA replication. NS3 contains a serine protease domain that requires NS2B as a cofactor at the N-terminus and an RNA-deconjugating enzyme domain at the C-terminus (Li and Kang, 2021). The N-terminus of NS5 has a methyltransferase structural domain, whereas the C-terminus has an RNA-dependent RNA polymerase (RdRp) domain (Hilgenfeld et al., 2018). Most of the antivirals under research are direct-acting, which target part of the virus directly, including RdRp, NS2B-NS3 protease, NS3 helicase and so on (Baz and Boivin, 2019; Yuan et al., 2020; Lin et al., 2023; Badshah et al., 2019).

EPA is a 20-carbon fatty acid including five double bonds, the first of which is located in the third carbon atom distal to the fatty acid tail (Yoshioka et al., 2022). It is a significant component of breast milk (Miles et al., 2021; Argaw et al., 2021), which plays an important role in protecting both term and preterm newborns from pathogenic infections (Dombrowska-Pali et al., 2024; Francese et al., 2023). It can reduce the viscosity of red blood cells, inhibit the platelet-mediated thrombosis, and prevent the formation and progression of atherosclerotic plaques(Calder, 2013). Due to health issues, numerous international health and food organizations have developed guidelines to ensure daily intake of fatty acids, especially EPA and DHA, indicating the safety of these fatty acids (Serefko et al., 2024). The ethyl ester of EPA (Vascepa^®^) has been approved by FDA in 2013 for reducing cardiovascular risk when added to a statin. It is probably appropriate for the development of prenatal medication. Studies have shown that some unsaturated fatty acids, such as arachidonic acid (AA), EPA, and docosahexaenoic acid (DHA), can inactivate enveloped viruses, such as SARS-CoV-2 (Das, 2021). However, studies investigating the effects of EPA on ZIKV infection are lacking. We have previously shown that alpha-linolenic acid (ALA) effectively inhibited ZIKV infection (Feng et al., 2023). Given that ALA is metabolized to EPA in the human body, we speculated that EPA might also exert antiviral activities against ZIKV. Therefore, in this study, we investigated the effects of EPA on ZIKV infection and explored the underlying mechanisms.

## 2 Materials and methods

### 2.1 Cells, plasmids, viral strains, and reagents

Vero cells (derived from the kidney of an African green monkey), Madin-Darby canine kidney (MDCK) cells, BHK21 cells (derived from the kidney of a Syrian baby hamster), and HEK-293T cells (derived from a human embryonic kidney) were grown in Dulbecco’s modified Eagle’s medium (DMEM) (Gibco, United States) supplemented with 10% fetal bovine serum (ExCell Bio, China) and 1% penicillin/streptomycin (Invitrogen, United States).

The ZIKV clinical isolate z16006 (GenBank: KU955589.1) was generously gifted by Prof. Changwen Ke (Guangdong Provincial Centre for Disease Control and Prevention). The New Guinea C strain of dengue virus type 2 (DENV-2) was generously gifted by Prof. Xiaoguang Chen (School of Public Health, Southern Medical University). The influenza A virus strain A/WSN/1933 (H1N1) was generously gifted by Prof. Zifeng Yang (State Key Laboratory of Respiratory Diseases, Guangzhou Medical University). A plasmid expressing ZIKV NS2B-NS3 protease was kindly gifted by Prof. Haitao Yang (ShanghaiTec University). EPA (≥98% purity) was purchased from Xianding Biotechnology Co., Ltd. (Shanghai, China). Etoposide erythromycin (Ery-Est) was purchased from Zhongcheng Jinnian Science and Technology Co., Ltd. (Beijing, China). And epigallocatechin gallate (EGCG) was purchased from Beijing Xinshengbaitai Technology Co., Ltd. (Beijing, China).

### 2.2 MTT assay

The cytotoxicity of EPA was determined using 3-(4,5-dimethylthiazolyl-2)-2,5-diphenyltetrazolium bromide (MTT) assay, which was based on the principle that living cells can reduce MTT to blue-violet crystals. Vero, BHK and MDCK cells were exposed to serial dilutions of EPA for 96 h and subsequently treated with 0.5-mg/mL MTT at 37°C for 4 h. Thereafter, the medium was aspirated and discarded, followed by the addition of dimethylsulphoxide (DMSO) to the cells. After 15 min, absorbance (λ = 570 nm) was measured using an enzyme marker (Tecan, Switzerland) and cell viability was calculated.

### 2.3 TCID50 endpoint titration

Infectious viral titers were determined using 50% tissue culture infectious dose (TCID_50_), an endpoint dilution assay. Briefly, a total of 1 × 10^3^ Vero cells were seeded in each well of a 96-well plate for 12 h. Subsequently, the cells were incubated with serially diluted viral reservoir solutions for 4–6 days. The cells were observed daily under a microscope for cellular lesions until no newly infected cells were observed. The TCID_50_ value was calculated using a method reported by Reed and Muench (Reed and Muench, 1938). The multiplicity of infection (MOI) of viral inoculation was defined as the number of viral particles per cell inoculated. MOI = (Viral titer × Inoculation volume)/Number of cells.

### 2.4 Cell-based immunodetection

The protein expression of ZIKV NS1 was determined via cell-based immunodetection according to a previously reported method (Zou et al., 2020). Briefly, cells were infected with ZIKV (100 TCID_50_) for 4 days, washed with PBS, and fixed with 4% paraformaldehyde (PFA) at 25°C for 20 min. The fixed cells were incubated with 100% pre-cooled methanol at 4°C for 5 min and washed with PBS. The cells were blocked with PBS containing 2% skimmed milk at 4°C for 1 h and washed with PBS containing 0.3% (v/v) Tween 20 (3 × 5 min). The cells were incubated with a primary antibody against ZIKV E or NS1 (1:1000) (BioFront Technologies, United States) at 37°C for 1 h, followed by incubation with a horseradish peroxidase (HRP)-labeled secondary antibody (1:2000) (Cell Signalling Technology, United States). Subsequently, the cells were washed with a wash buffer (5 × 5 min) and incubated with 3,3′,5,5′-tetramethylbenzidine (TMB) as a peroxidase substrate for 5 min in the dark. Finally, H_2_SO_4_ (1 mM) was added to each well to terminate the reaction and the optical density at 450 nm was measured using a GeniosPro microplate reader (Tecan, United States).

### 2.5 Quantitative reverse transcription PCR (qRT-PCR)

Viral RNA was extracted from cell pellets using a viral RNA extraction kit (Promega, United States) according to the manufacturer’s instructions. The extracted RNA was reverse transcribed to complementary deoxyribonucleic acid (cDNA) using a SuperScript-III kit (Takara, Japan). Subsequently, quantitative PCR was performed on a LightCycler^®^ 480 system (Roche, United States) using SYBR Green Ab Taq qPCR Mix (Biotech Inc., China) and appropriate primers. The relative mRNA levels of target viral genes were measured using the 2^−ΔΔCT^ method and normalized to that of GAPDH. Ery-Est (20 μM) was set as a positive control. The primer sequences used for PCR are as follows: ZIKV forward, 5′- CVGACATGGCTTCGGACAGY-3′; ZIKV reverse, 5′- CCCARCCTCTGTCCACYAAYG-3′; ZIKV E forward, 5′- GGTGGGACTTGGGTTGATGT-3′; ZIKV E reverse, 5′- ATG​TCA​CCA​GGC​TCC​CTT​TG-3′; ZIKV NS1 forward, 5′- ACC​AGA​GAG​GGC​TAC​AGG​AC-3′; ZIKV NS1 reverse, 5′- TTA​GCC​TGG​AAC​GAC​AGT​GG-3′; HSV-1 VP16 forward, 5′- TTG​ACT​GCC​TCT​GTT​GCG​AC-3′; HSV-1 VP16 reverse, 5′- ATG​TGG​TTT​AGC​TCC​CGC​AG-3′; HSV-1 gB forward, 5′- AAC​GCG​ACG​CAC​ATC​AAG-3′; HSV-1 gB reverse, 5′- CTG​GTA​CGC​GAT​CAG​AAA​GC-3′; GAPDH forward, 5′- TTG​CAT​CGC​CAG​CGC​ATC-3′; GAPDH reverse, 5′- TCG​CCC​CAC​TTG​ATT​TTG​GA -3′.

### 2.6 Western blotting

Cells were collected and lysed with radioimmunoprecipitation assay (RIPA) buffer to extract total proteins. A bicinchoninic acid (BCA) assay kit (KeyGEN, China) was used to quantify the extracted proteins. The proteins were separated on a 10% sodium dodecyl sulfate–polyacrylamide gel and transferred to a polyvinylidene difluoride membrane (Millipore, United States) using the wet transfer method. Non-specific binding sites were blocked by incubating the membrane with tris-buffered saline (TBS) containing 5% fat-free milk and 0.1% Tween 20 for 1 h at room temperature. Subsequently, the membrane was incubated with a primary antibody and a horseradish peroxidase-conjugated secondary antibody. EGCG (20 μM) was set as a positive control. Protein bands were detected using enhanced chemiluminescence (ECL), and images were captured via a FluorChem R imaging system.

### 2.7 Assay for virus binding

Vero cells were seeded in a 6-well plate and cultured overnight. EPA was incubated with ZIKV (MOI = 2.5) at 37°C for 0.5 h, and the mixture was subsequently added to the cultured cells. After incubation at 4°C for 1 h, the cells were washed and collected, followed by viral RNA extraction and qRT-PCR analysis.

### 2.8 Assay for virus adsorption

Vero cells were seeded in a 6-well plate and cultured overnight. EPA was mixed with viral stock solutions separately (MOI = 1), and cultured cells were exposed to the mixture at 4°C for 1 h. Subsequently, the culture supernatant was discarded and the cells were washed to remove free viral particles. The cells were maintained in DMEM at 37°C for 72 h. Finally, viral RNA was extracted, reverse transcribed, and subjected to qRT-PCR analysis.

### 2.9 Assay for virus entry

Vero cells were seeded in a 6-well plate and cultured overnight. The cells were incubated with ZIKV (MOI = 1) at 4°C for 1 h, followed by the removal of the viral solution. The cells were washed and incubated with fresh medium containing serially diluted EPA at 37°C for 1 h, followed by the removal of the EPA-containing medium. The cells were washed with PBS and maintained in DMEM at 37°C for 72 h. Finally, viral RNA was extracted, reverse transcribed, and subjected to qRT-PCR analysis.

### 2.10 Time-of-addition assay

Time-of-addition assay was performed to identify the phase of the ZIKV replication cycle that was affected by EPA. Briefly, cells were treated with virus–EPA mixtures (MOI = 1) pre-incubated for 0, 5, 10, 20, and 40 min, or EPA was added to cells at 0, 0.5, 1, 2, and 4 h after 1 h post infection (hpi). The working concentration of EPA was 10 μM. The cells were maintained at 37°C for 24 hpi. Thereafter, the cells were collected and viral RNA was extracted for qRT-PCR analysis.

### 2.11 RNase digestion assay

EPA was serially diluted and incubated with ZIKV (700 TCID_50_) at 37°C for 2 h. Subsequently, the mixture was incubated with micrococcal nuclease at 37°C for 1 h (Xiong et al., 2021). After the reaction was terminated by EGTA (10 mM), viral RNA was extracted using an EasyPure viral RNA extraction kit (TransGen Biotech, China), reverse transcribed, and subjected to qRT-PCR analysis.

### 2.12 ZIKV NS2B-NS3 protease inhibition assay

Fluorescence resonance energy transfer (FRET) spectroscopy was used to assess whether EPA could inhibit NS2B-NS3 protease activity. ZIKV NS2B-NS3 protease was produced in *E. coli* Rosetta (DE3) and purified using a Superdex 200 PG column (GE Healthcare, United States) as described previously (DiNunno et al., 2020). The purified NS2B-NS3 protease (0.1 μM) was incubated with or without EPA at 37°C for 30 min, followed by the drop-wise addition of Bz-Nle-Lys-Arg-Arg-AMC (substrate) at a concentration of 100 μM to initiate the reaction. After 10 min of incubation, fluorescent signals were detected at 360/450 nm using a microplate reader. Aprotinin at a concentration of 1 μM was used as a positive control.

### 2.13 DENV inhibition assay

BHK-21 cells were seeded in a 12-well plate and cultured overnight. The next day, diluted EPA was incubated with dengue virus for 10 min and the mixture was added to the cells (MOI = 1). After incubation for 1 h at 37°C, the virus-containing supernatant was discarded, and fresh medium without FBS containing 0.6-μg/mL L-1-toluenesulfonylamino-2-phenylethylchloromethylketone (TPCK) (Sigma Aldrich, Missouri, United States) was added to the cells. The antiviral efficacy of EPA was examined by the above MTT assay.

### 2.14 HSV-1 inhibition assay

Vero cells were inoculated into a 6-well plate, cultured overnight and washed with PBS. Then the cells were infected with a mixture of pre-cultured EPA and HSV-1 (MOI = 1). After incubation at 37°C for 1 h, the virus-containing supernatant was replaced by fresh medium and cultured for 24 h. The expression of herpes simplex virus envelope glycoprotein D (gD) was detected by Western blotting, and the transcription of gB and VP16 mRNA was detected by qRT-PCR.

### 2.15 Influenza A virus inhibition assay

MDCK cells were seeded in a 12-well plate and cultured overnight. The following day, serially diluted EPA was incubated with influenza A virus for 10 min and the mixture was subsequently added to the cells. After 1 h of incubation at 37°C, the viral solution was discarded and the FBS-free fresh medium containing TPCK (0.6 μg/mL) was added to the cells. After 48 h, the antiviral effects of EPA were examined via MTT assay.

### 2.16 Animal experiments

Male or female C57 mice aged 8–10 weeks were randomly assigned to four groups (n = 5). After 12 h of fasting, the mice were administered EPA at concentrations of 10, 50, and 100 mg/kg of body weight or a vehicle (saline; control) via gavage. The treatments were administered for 2 consecutive days, and the mortality of mice was observed. Orbital blood and urine samples were collected on days 1, 2, 3, 5, and 7 after the last administration. Serum levels of alanine aminotransferase (ALT) and urinary levels of creatinine (CRE) were measured using corresponding kits (NJJCBIO, Nanjing, China) according to the manufacturer’s protocols. Liver and kidney tissues were collected from each mouse for hematoxylin–eosin (HE) staining. The tissues were fixed in 4% paraformaldehyde, embedded in paraffin, and sectioned at a thickness of 5 μm. HE staining was performed to observe histopathological changes in the mesenchyme and parenchyma under a light microscope.

### 2.17 Statistical analysis

GraphPad Prism 9.0 was used to produce graphs and perform all statistical analyses. The half-maximal inhibitory concentration (IC_50_) and half-maximal cytotoxic concentration (CC_50_) were determined via non-linear regression analysis of concentration–effect curves and expressed as the mean ± standard deviation (SD). Data were compared using one-way ANOVA followed by Bonferroni’s multiple comparison test. A p-value of <0.05 was considered significant (*, P < 0.05; **, P < 0.01; ***, P < 0.001; ****, P < 0.0001).

## 3 Results

### 3.1 Toxic effects of EPA *in vitro* and *in vivo*

**FIGURE 1:**
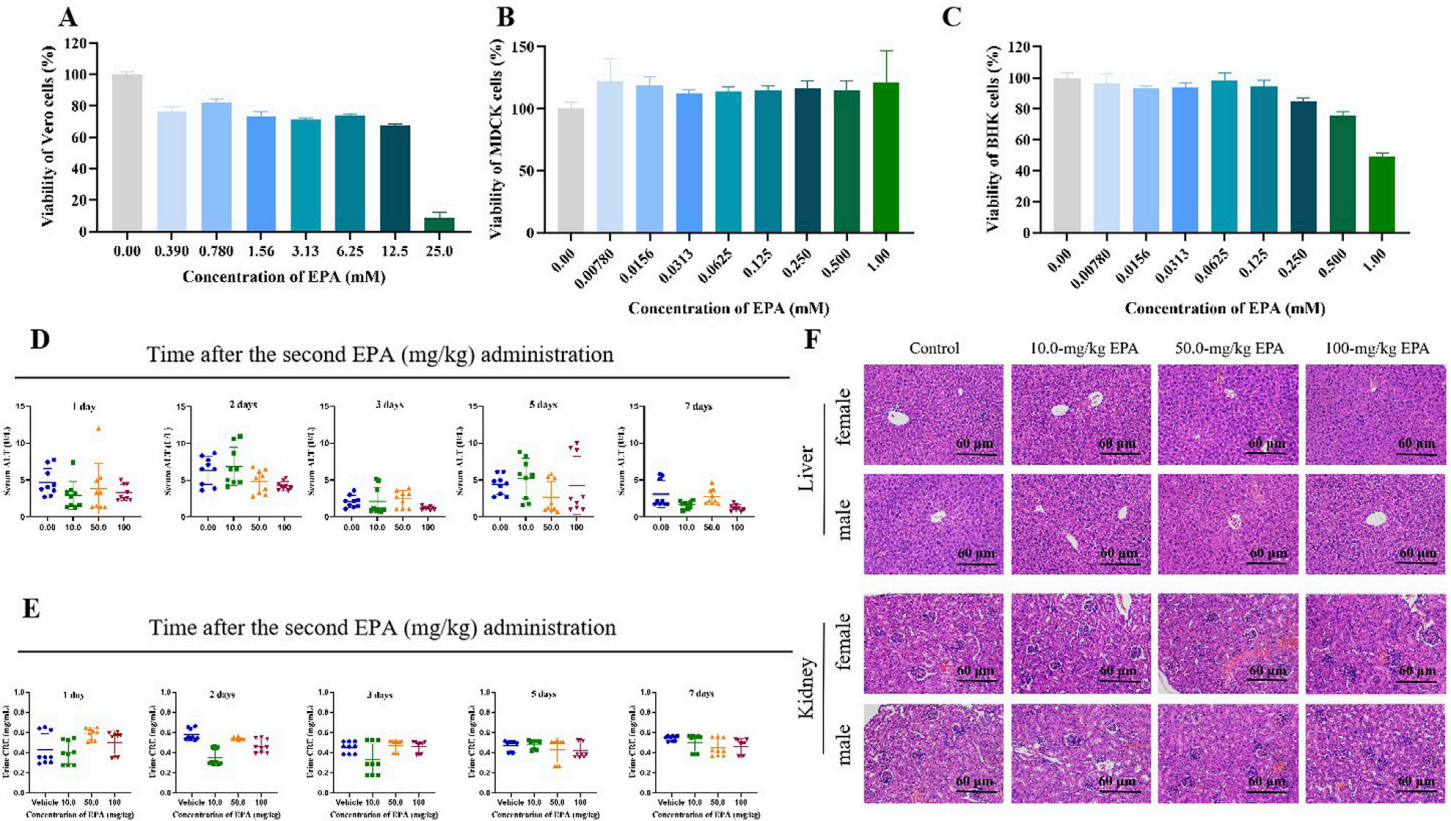
Toxicity of EPA *in vitro* and *in vivo*. Effects of EPA on the viability of Vero **(A)**, MDCK **(B)**, and BHK-21 **(C)** cells. All experiments were repeated three times. **(D)** Serum ALT levels in mice were measured on days 1, 2, 3, 5, and 7 after the last administration of EPA (0–100 mg/kg) using an ALT assay kit. The error bars represent the SD of the mean. **(E)** Urinary CRE levels in mice were measured on days 1, 2, 3, 5, and 7 after the last administration of EPA (0–100 mg/kg) using a CRE assay kit. The error bars represent the SD of the mean. **(F)** Representative images of HE staining of liver and kidney tissues in each group.

MTT assay was used to evaluate the toxic effects of EPA on Vero, MDCK, and BHK-21 cells. EPA was added to the cells at increasing concentrations to determine the CC_50_ values. The results (Figures 1A, C) showed that the CC_50_ value of EPA in Vero cells was 10.18 ± 7.11 mM and those in MDCK and BHK-21 cells were >1 mM. These results indicated that EPA had extremely low cytotoxicities. Therefore, its toxic effects on cells could be neglected in subsequent experiments.

To evaluate the safety of EPA in animals, we randomly divided mice into four groups and administered EPA (10, 50, and 100 mg/kg of body weight; experimental groups) or saline (control group) via gavage for 2 days. Serum ALT and urinary CRE levels were measured to evaluate the hepatotoxicity and nephrotoxicity of EPA in mice. As shown in Figures 1D, E, no significant differences in ALT or CRE levels were observed between the experimental and control groups, indicating that EPA was neither hepatotoxic nor nephrotoxic in mice at the administered concentrations. Consistently, HE staining of liver and kidney tissues (Figure 1F) showed that EPA did not cause tissue damage in mice at the administered concentrations.

### 3.2 EPA suppressed ZIKV activity in a dose-dependent manner

**FIGURE 2:**
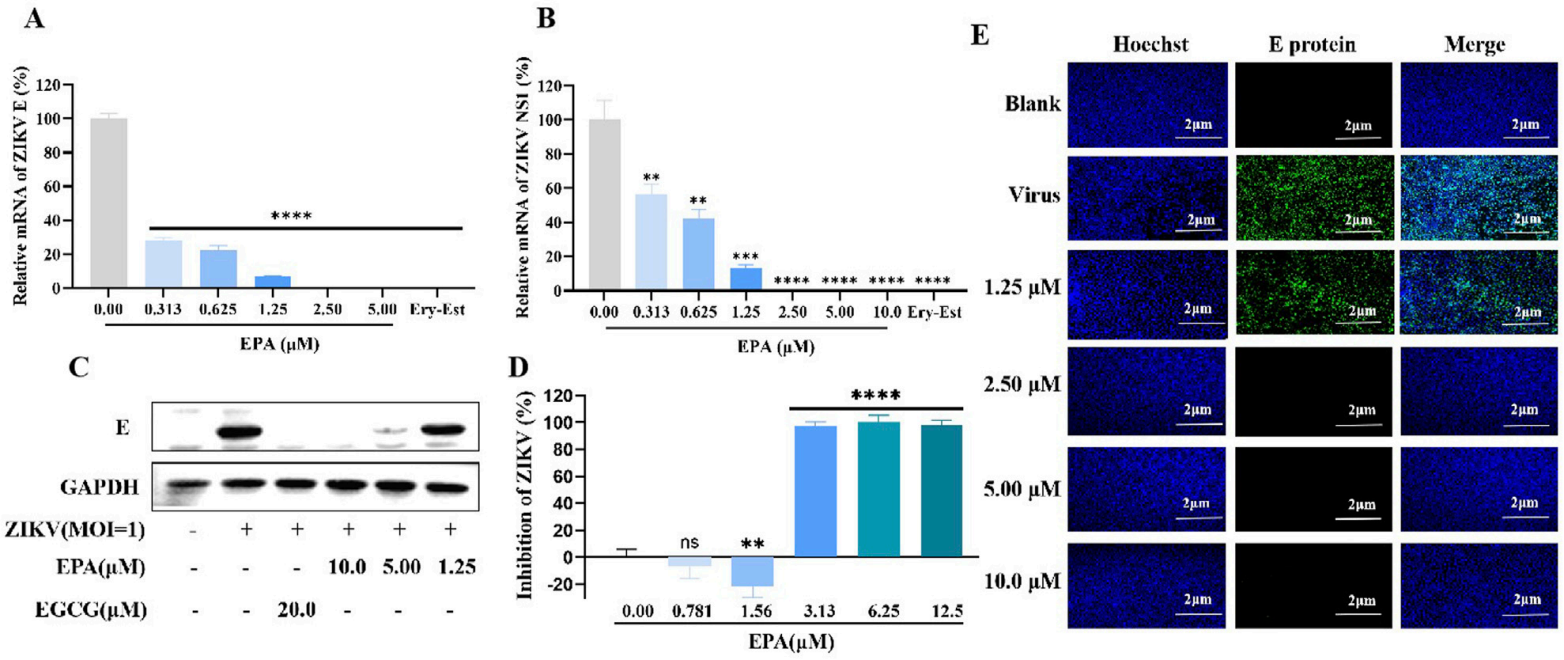
Inhibition of ZIKV activity by EPA *in vitro*. The mRNA levels of ZIKV E **(A)** and NS1 **(B)** was evaluated in ZIKV-infected Vero cells treated with EPA via qRT−PCR. **(C)** The protein expression of ZIKV E was evaluated in ZIKV-infected Vero cells treated with EPA via Western blotting. **(D)** ZIKV activity was evaluated in ZIKV-infected Vero cells treated with EPA via cell-based immunodetection assay. **(E)** ZIKV activity was evaluated in ZIKV-infected Vero cells treated with EPA via indirect immunofluorescence analysis. All experiments were repeated three times. *p < 0.05; **p < 0.01; ***p < 0.001; ****p < 0.0001; n. s = not significant.

The antiviral effects of EPA against ZIKV were examined using a set of assays. A pre-incubated mixture of ZIKV and serially diluted EPA was inoculated in Vero cells. After 1 h, the cells were washed, replenished with fresh medium, and cultured for 2 days. Subsequently, total cellular RNA was extracted and the relative mRNA levels of ZIKV E and NS1 were measured via qRT-PCR. As shown in Figures 2A, B, EPA decreased the mRNA levels of both ZIKV genes in a dose-dependent manner, with the IC_50_ value of EPA against ZIKV E being 0.42 ± 0.06 μM.

Consistently, EPA decreased the protein expression of ZIKV E in a dose-dependent manner (Figure 2C). In particular, EPA had significant inhibitory effects at concentrations of ≥5 μM. Cell-based immunodetection assay and indirect immunofluorescence assay also showed that EPA reduced the cytoplasmic expression of ZIKV E protein and ZIKV progeny viruses, respectively (Figures 2D, E). These preliminary results suggest that EPA exerts notable therapeutic effects against ZIKV infection.

### 3.3 EPA rapidly inhibited ZIKV activity and targeted the virus

**FIGURE 3:**
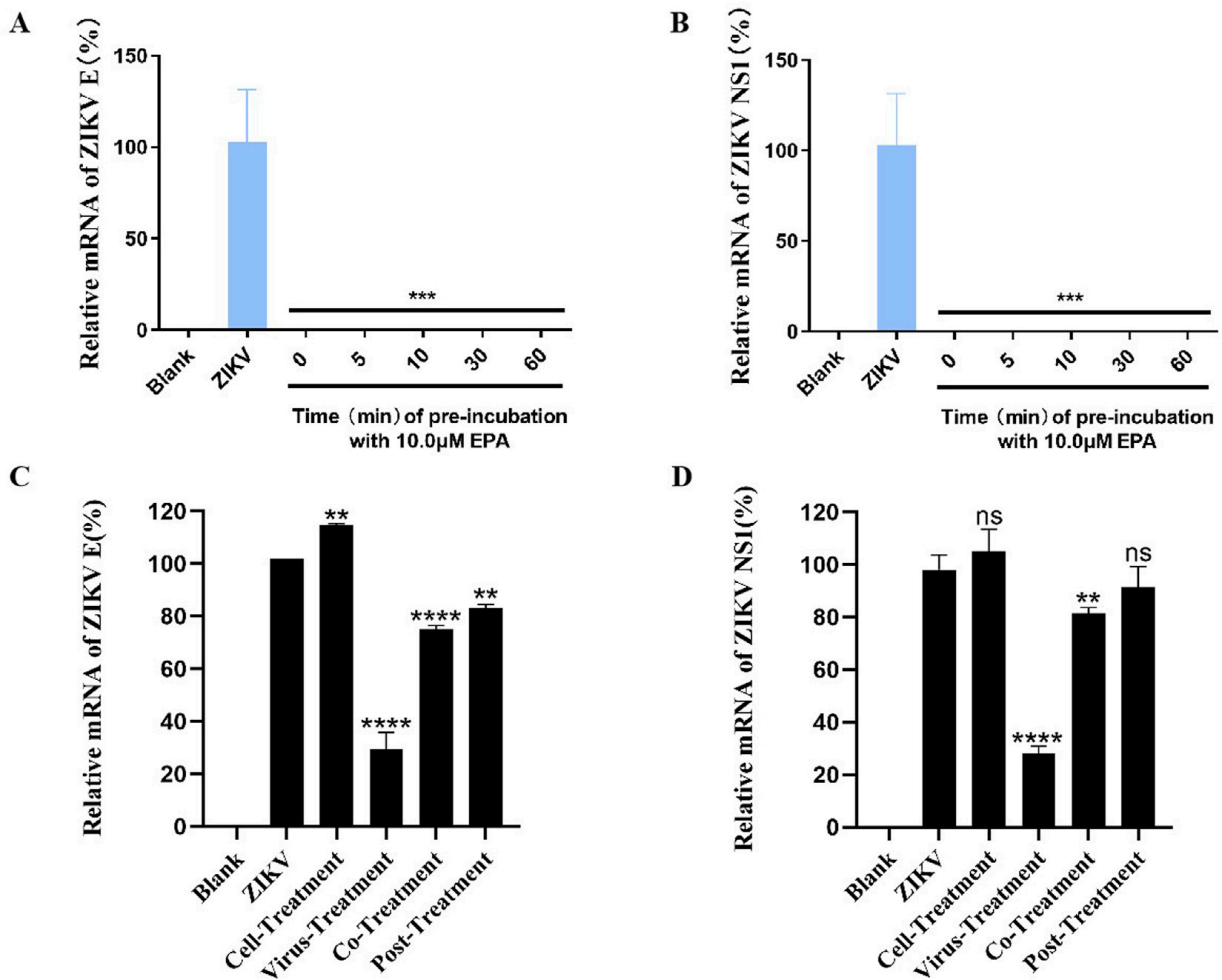
EPA rapidly inhibited ZIKV replication and targeted the virus. Determination of mRNA levels of ZIKV E **(A)** and NS1 **(B)** in cells infected with EPA–ZIKV mixture pre-incubated for 0, 5, 10, 30, and 60 min. Determination of the mRNA levels of ZIKV E **(C)** and NS1 **(D)** in cells, which were divided into four groups according to the treatment of EPA and ZIKV. Cell-treatment group: EPA was incubated with Vero cells for 1 h before ZIKV infection. Virus-treatment group: ZIKV was pre-incubated with EPA for 30 min and subsequently inoculated into cells. Co-treatment group: EPA and ZIKV were simultaneously inoculated into cells. Post-treatment group: Cells were incubated with ZIKV for 1 h and washed before being treated with EPA. All experiments were repeated three times. *p < 0.05; **p < 0.01; ***p < 0.001; ****p < 0.0001; n. s = not significant.

To assess the time required by EPA to exert antiviral effects, ZIKV was pre-incubated with or without EPA for various durations, ranging from 0 to 60 min, and the mixture was subsequently added to Vero cells. After 1 h of incubation, the supernatant was discarded and fresh medium was added. After 48 h, the cells were harvested and the mRNA levels of ZIKV E and NS1 were evaluated via qRT-PCR assay. The results (Figures 3A, B) showed that 100% inhibition of viral activity was achieved even without pre-incubation, suggesting that EPA rapidly inhibited ZIKV replication.

To investigate whether EPA targeted the virus or the cell, we divided Vero cells into four groups according to the treatment of EPA and ZIKV. In the cell-treatment group, the cells were incubated with EPA (10 μM) for 1 h, washed, and incubated with ZIKV (MOI = 1) for 1 h. In the virus-treatment group, EPA was pre-incubated with ZIKV for 30 min and the cells were subsequently incubated with the mixture for 1 h. In the co-treatment group, the cells were co-incubated with EPA and ZIKV for 1 h. In the post-treatment group, the cells were incubated with ZIKV for 1 h, washed then followed by the addition of EPA. At 24 hpi, all cells were harvested and the mRNA levels of ZIKV E and NS1 were evaluated via qRT-PCR. As shown in Figures 3C, D, EPA significantly inhibited ZIKV activity in the virus-treatment group, indicating that it targets the virus instead of the cell.

### 3.4 EPA disrupted the binding, adsorption and entry stages of ZIKV life cycle

**FIGURE 4:**
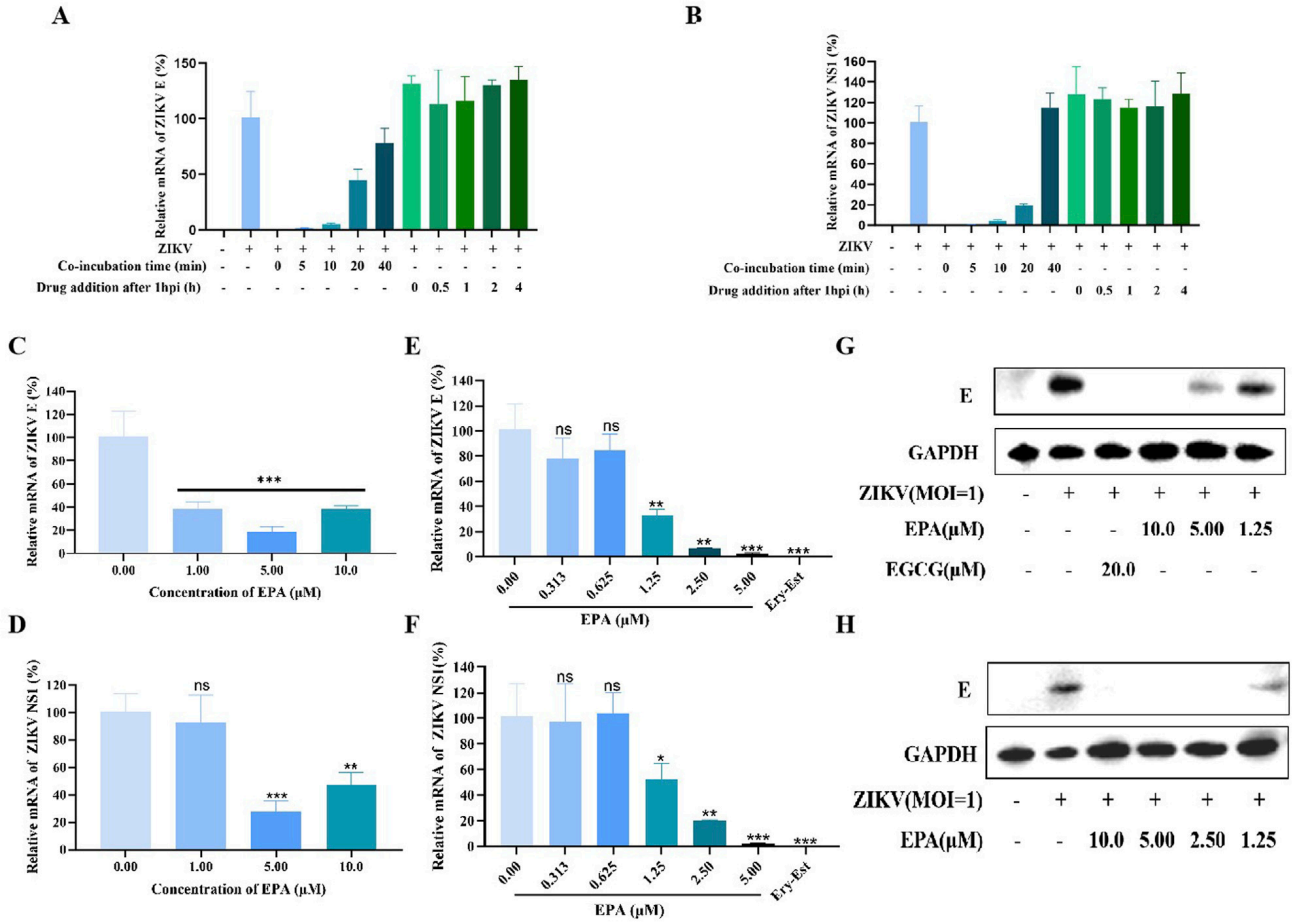
EPA disrupted the early stages of the ZIKV replication cycle. **(A, B)** Results of time-of-addition assay. The mRNA levels of ZIKV E **(A)** and NS1 **(B)** were evaluated via qRT-PCR. EPA was added at 0, 5, 10, 20, and 40 min after ZIKV infection or at 0, 0.5, 1, 2, and 4 h after 1 h of ZIKV infection. The effects of EPA on the binding of ZIKV to host cells **(C, D)**, its adsorption on host cells **(E–G)**, and its entry into host cells **(H)** are shown. The effects of EPA on the mRNA **(C–F)** and protein **(G, H)** expression of ZIKV E are shown. All experiments were repeated three times. *p < 0.05; **p < 0.01; ***p < 0.001; ****p < 0.0001; n. s = not significant.

Time-of-addition assay was used to investigate the mechanism of EPA against ZIKV. Briefly, cells were incubated with ZIKV for 0, 5, 10, 20, or 40 min, followed by the addition of EPA (10 μM), or cells were washed at 1 hpi and the time was recorded as 0 h, followed by the addition of EPA at 0, 0.5, 1, 2, or 4 h. After 24 h of incubation, cellular RNA was isolated for qRT-PCR. As shown in Figures 4A, B, the earlier the addition of EPA after ZIKV infection, the better the antiviral effects. However, EPA did not affect cells that had been infected with ZIKV for 1 h. These results indicated that EPA interfered with the early stages of the ZIKV life cycle.

The early stages of the viral life cycle include binding, adsorption, and entry. To assess the effects of EPA on the binding of ZIKV to host cells, Vero cells were treated with pre-incubated EPA–ZIKV mixtures at 4°C for 1 h. Subsequently, cellular RNA was isolated to evaluate the mRNA levels of ZIKV E and NS1 via qRT-PCR. As shown in Figures 4C, D, EPA inhibited the binding of ZIKV to host cells at the administered concentrations.

To assess the effects of EPA on the adsorption of ZIKV onto host cells, EPA and ZIKV were simultaneously added to cells, followed by 1 h of incubation at 4°C. At this stage, the virus could only be adsorbed on the surface of the cells but could not enter them. EGCG or Ery-Est at 20 μM was used as the positive control. After incubation, the medium was replaced with fresh medium and the cells were maintained at 37°C for 72 h. Thereafter, total RNA and protein were extracted from cells for qRT-PCR and Western blotting, respectively. As shown in Figures 4E–G, EPA inhibited ZIKV E and NS1 at both mRNA and protein levels in a dose-dependent manner. The inhibition rate reached approximately 100% when the concentration of EPA was >5 μM. These results indicated that EPA effectively prevented the adsorption of ZIKV onto host cells.

To assess the effects of EPA on the entry of ZIKV into host cells, the cells were incubated with ZIKV at 4°C for 1 h. After removing the virus-containing medium, the cells were rinsed with PBS and incubated with EPA at 37°C for 1 h. During this process, if EPA prevented ZIKV from entering host cells, the viral protein content should be lower than that in the negative-control group. As shown in Figure 4H, EPA also prevented the entry of ZIKV into host cells.

These results collectively suggest that EPA inhibits the binding, adsorption and entry of ZIKV in the early stages of its life cycle.

### 3.5 EPA disrupted the integrity of ZIKV membrane probably by binding to E protein

**FIGURE 5:**
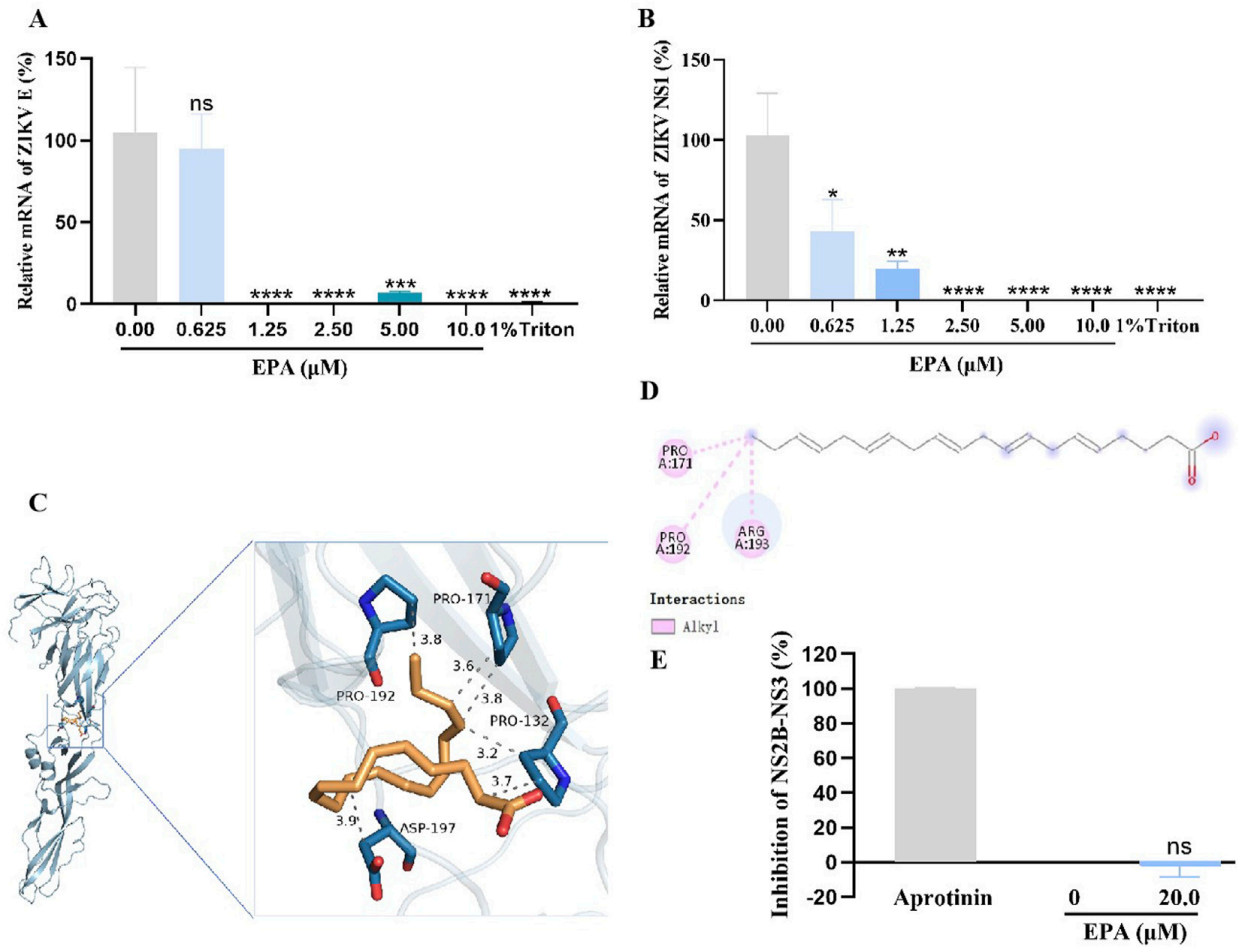
EPA destroyed the ZIKV envelope by binding to E protein. The mRNA levels of ZIKV E **(A)** or the whole viral genome **(B)** were evaluated after performing the micrococcal nuclease digestion assay. The three-dimensional **(C)** and two-dimensional **(D)** diagrams of the EPA–ZIKV docked complexes show interactions between EPA and amino acid residues of ZIKV E protein. **(C)** In the 3D diagram, the gray dotted lines represent hydrophobic bonds. **(D)** In the 2D diagram, the pink dotted lines represent hydrophobic bonds. **(E)** The effects of EPA on ZIKV NS2B-NS3 protease were assessed via FRET spectroscopy. All experiments were repeated three times. *p < 0.05; **p < 0.01; ***p < 0.001; ****p < 0.0001; n. s = not significant.

The abovementioned results suggest that EPA interferes with the replication of ZIKV by directly acting on it. Therefore, we speculated that EPA might target the viral envelope. To verify this speculation, we performed RNase digestion assay using micrococcal nuclease, which can degrade viral RNA in the absence of a complete envelope. As shown in Figures 5A, B, EPA decreased the mRNA levels of ZIKV E and the whole viral genome in a concentration-dependent manner, indicating that it disrupted the viral envelope. The effects of EPA at 2.5 μM were comparable to those of 1% Triton (positive control). These results indicated that EPA exerted antiviral effects by disrupting the envelope of ZIKV.

Furthermore, the binding affinity between EPA and ZIKV E protein was predicted via molecular docking using the AutoDock Vina software (version 1.1.2). The conformation of the EPA–ZIKV complex with the strongest binding affinity is presented in Figures 5C, D. The binding affinity (KD) of EPA for ZIKV E protein was estimated to be −3.6 kcal/mol. In this docked complex, EPA formed two hydrophobic bonds with the amino acid residue PRO 171, with bond lengths of 3.6 Å and 3.8 Å; two hydrophobic bonds with the amino acid residue PRO 132, with bond lengths of 3.2 Å and 3.7 Å; and two hydrophobic bonds with the amino acid residues PRO 192 and ASP 197, with bond lengths of 3.8 Å and 3.9 Å, respectively (Figure 5C). Additionally, the 2D diagram showed that EPA formed a hydrophobic bond with the amino acid residue ARG 193 (Figure 5D).

The NS2B-NS3 protease plays an important role in the early stages of ZIKV replication. We also examined the effects of EPA on the activity of ZIKV NS2B-NS3 protease via FRET spectroscopy. As shown in Figure 5E, the protease activity was completely blocked by aprotinin (positive control), indicating the validity of the experiment. However, EPA did not have significant inhibitory effects on ZIKV NS2B-NS3 protease when compared with the negative-control group. These results indicate that EPA exerts antiviral effects by disrupting the envelope of ZIKV.

### 3.6 EPA inhibited the activities of DENV, HSV-1, and influenza virus

**FIGURE 6:**
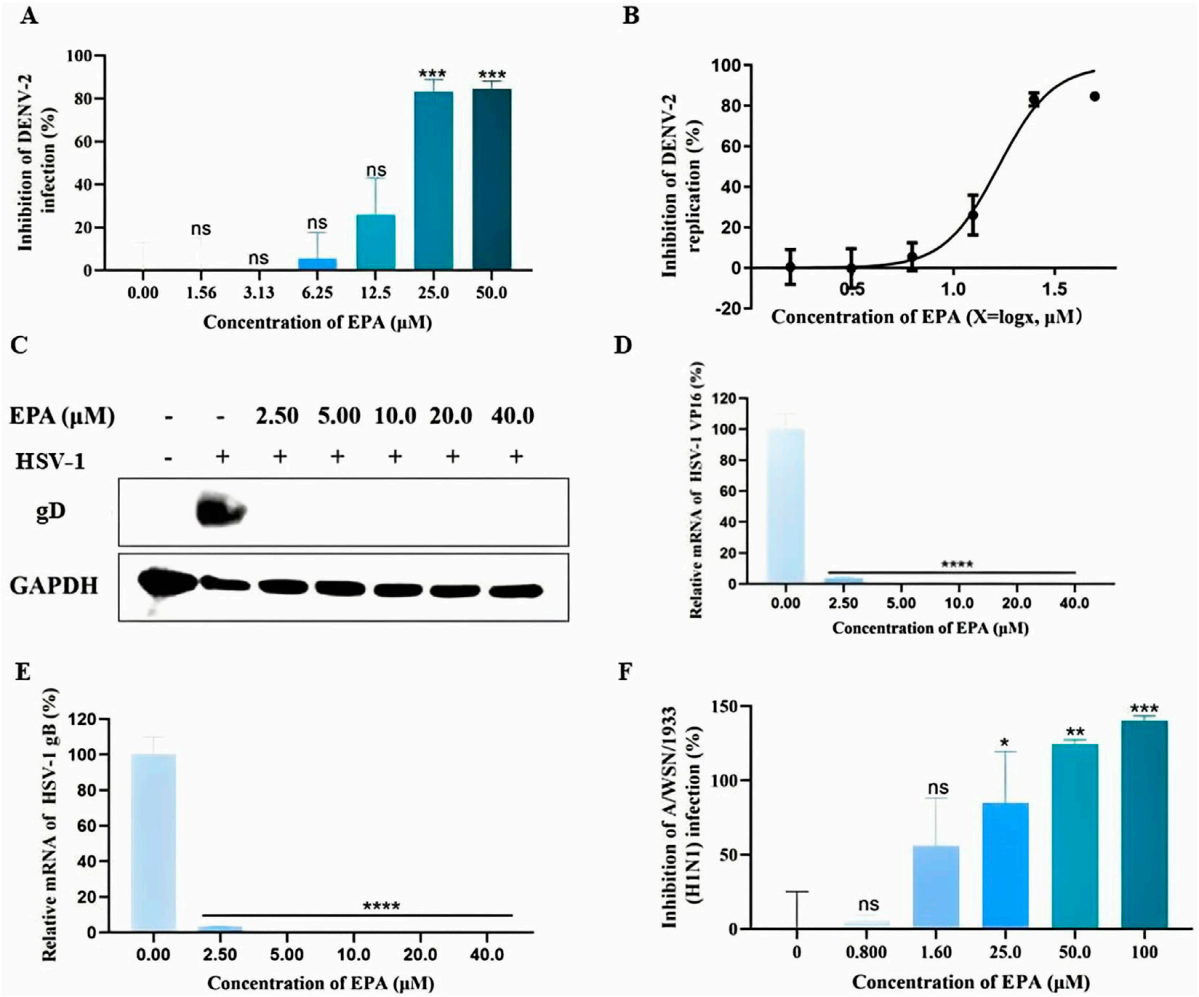
EPA exhibited broad-spectrum antiviral activities. **(A)** The effects of EPA on dengue virus replication were evaluated via MTT assay. **(B)** Semi-logarithmic coordinate graph demonstrating the inhibitory effects of EPA on dengue virus replication. **(C)** The effects of EPA on HSV-1 gD protein were evaluated via Western blotting. **(D)** The effects of EPA on HSV-1 VP16 mRNA were evaluated via qRT-PCR. **(E)** The effects of EPA on HSV-1 gB mRNA were evaluated via qRT-PCR. **(F)** The effects of EPA on influenza A virus were evaluated via MTT assay. All experiments were repeated three times. *p < 0.05; **p < 0.01; ***p < 0.001; ****p < 0.0001; n. s = not significant.

To examine the effects of EPA on DENV replication, BHK-21 cells were infected with a mixture of pre-incubated EPA and DENV-2. After 1 h of incubation, the viral solution was replaced with fresh medium and the cells were cultured for 48 h. Subsequently, MTT assay was performed to determine the cell survival rate. As shown in Figures 6A, B, EPA inhibited the replication of DENV-2 in a concentration-dependent manner.

To examine the effects of EPA on HSV-1, the content of envelope glycoprotein (gD) was evaluated via Western blotting. As shown in Figure 6C, EPA administered at concentrations of 2.5–40 μM successfully inhibited HSV-I replication. In addition, EPA completely inhibited the mRNA levels of HSV-Ⅰ VP16 and gB at concentrations of >5 μM (Figures 6D, E), which was consistent with the results of Western blotting (Figure 6C).

To examine the effects of EPA on influenza A virus replication, MDCK cells were infected with a pre-incubated mixture of EPA and influenza virus for 1 h. The viral solution was subsequently replaced with fresh medium containing TPCK-trypsin, and cell survival was assessed via MTT assay after 48 h. As shown in Figure 6F, EPA inhibited the replication of influenza A virus in a concentration-dependent manner.

Altogether, these results indicate that EPA effectively inhibits the replication of dengue virus, HSV-1, and influenza A virus.

## 4 Discussion

Human breast milk contains essential nutrients and vitamins that nourish and protect infants. In a recent study, we showed that stored breast milk produced potent anti-ZIKV factors (Conzelmann et al., 2019). The antiviral activity of breast milk is associated with the endogenous lipase-dependent production of free fatty acids (Francese et al., 2020; Wedekind and Shenker, 2021). Free fatty acids, including arachidonic, oleic, and linoleic acids, disrupt the viral lipid envelope, consequently suppressing viral replication and alleviating infection (Pfaender et al., 2013).

Unsaturated fatty acids, a class of fatty acids, are essential nutrients for human body and play important roles in brain development and maintenance of normal brain function. Approximately 80 years ago, omega-3 fatty acids, which are polyunsaturated fatty acids (PUFAs), were found to be essential for normal growth and health (Ali and Szabó, 2023). As components of the cell membrane, omega-3 fatty acids can regulate membrane fluidity and complex assembly in lipid rafts (Gutiérrez et al., 2019; Xie et al., 2024). In addition, they can increase cell membrane stability, modulate immune function, and block excessive inflammatory responses (Mayer and Seeger, 2008). Omega-3 fatty acids found in fish oil are mainly composed of EPA and DHA. EPA and DHA can alter the membrane lipid composition and function of T lymphocytes, increase the stability of cell membranes, influence immune function, and inhibit hepatitis C virus replication (Kar et al., 2023; Liu et al., 2010). Furthermore, PUFAs may reduce the risk of viral infections, such as SARS-CoV-2 infection, and may be used in prophylactic adjuvant therapy (Mazidimoradi et al., 2022).

This study showed that EPA inhibited the expression of ZIKV proteins and destroyed the viral envelope in a concentration-dependent manner. Time-of-addition assay showed that the earlier EPA was added, the better antiviral effects were achieved. However, after virus entry into host cells, treatment with EPA had no antiviral effects. EPA effectively inhibited the binding of ZIKV to host cells, its adsorption on the cell surface, and its entry into the cells. In the typical flavivirus life cycle, viral proteins are translated 1–5 hpi, viral RNA synthesis occurs ≥5 hpi, and the release of offspring virus occurs ≥12 hpi (Rausch et al., 2017; Chen H. et al., 2020). Therefore, if cells have been infected with ZIKV before treatment with EPA, part of viral RNA will have been translated into polyproteins and EPA will not work against this viral population.

In Figures 3C, D, there was a slight increase in the cell-treatment group compared with ZIKV group. EPA belongs to the essential fatty acids, which participate in phospholipid synthesis, and the cell membrane is composed of phospholipid. Thus EPA plays an important role in the formation of cell membranes and their function. The glucose-stimulated cholesterol crystalline domain formation, which was associated with less lipid oxidation, was concentration-dependently inhibited by EPA (Sherratt et al., 2021). And in human endothelial cells, EPA influences the protein distribution of caveolae lipid rafts in the membrane and subcellular fraction, as well as its acyl chain composition. (Li et al., 2007; Mason et al., 2018). For virus infection, the first step is the binding of virus to the receptors on the cell surface. Maybe the preincubated EPA increased the amount of receptors which can bind more virus. There is another possibility that EPA modifies the charge of the membrane and increases the binding of virus to the cell surface. And in Figures 3C, D, there was a decrease in the post-treatment group compared with ZIKV group. The cells were firstly infected with ZIKV for 1 h, washed then followed by the addition of EPA. Maybe after washing, there was still some virus bound to the cells. EPA inhibited their entry into the cells, thus the infection was partly restrained.

Unsaturated fatty acids, such as arachidonic acid (AA), EPA, and DHA, can inhibit the activity of enveloped viruses and the proliferation of diverse microorganisms. We performed RNase digestion assay and confirmed the disruption of the viral envelope by EPA. The docking method predicted the binding affinity between EPA and ZIKV E protein, but it needs to be validated in the cell or mouse models. In the envelope, both receptor attachment proteins and membrane fusion proteins are encoded by the virus (Li et al., 2021). So other proteins should also be taken into account. And some dotted materials such as cationic gemini amphiphiles were used to regulate the membrane charge and bilayer packing, then alter the membrane fluidity, and ultimately play a role in the adhesion and internalization abilities of giant unilamellar vesicles to the *E. coli* (Dai et al., 2023). Similarly, the viral membrane is a lipid bilayer that comes from host cells. Besides binding to the proteins directly, another possible mechanism of EPA may be destroying the lipid bilayer through charge interaction.

Alpha-linolenic acid (ALA) is a major dietary source of EPA, as ALA is biochemically converted to EPA (Lin et al., 2010). We have previously validated the inhibitory effects of ALA on ZIKV replication. In this study, we found that EPA, a metabolite of ALA, had similar inhibitory effects on ZIKV replication. The IC_50_ and CC_50_ of ALA are 4.29 ± 0.61 μM and 5.94 ± 0.67 mM, respectively (Feng et al., 2023). While those of EPA are 0.42 ± 0.06 μM and 10.18 ± 7.11 mM, respectively. EPA is more efficient and safer than ALA. Therefore, EPA is a promising drug candidate for treating ZIKV infection.

Furthermore, this study showed that EPA inhibited the replication of DENV-2, HSV-1, and influenza virus, suggesting that EPA has broad-spectrum antiviral activities. Mechanistically, EPA exerted antiviral effects by disrupting the integrity of the viral envelope, which is a common mechanism of several existing antiviral drugs (Xiong et al., 2021; Wang et al., 2019; Chen Y. et al., 2020). However, whether this mechanism works for all enveloped viruses warrants further investigation. EPA serves as the primary substrate for the production of anti-inflammatory cytokines. And the intake of EPA and DHA in BRC patients may contribute to preservation of optimal body weight and nutritional condition, elimination of proinflammatory cytokines (Arsic et al., 2023). Whether EPA influences the virus-induced inflammatory pathways worth exploring. As an essential fatty acid for normal human growth and health, EPA has advantages over existing antiviral drugs in terms of safety and availability. In conclusion, EPA is an ideal candidate for the development of broad-spectrum antiviral drugs.

## Data Availability

The raw data supporting the conclusions of this article will be made available by the authors, without undue reservation.
